# Impact of SCHC Compression and Fragmentation in LPWAN: A Case Study with LoRaWAN

**DOI:** 10.3390/s20010280

**Published:** 2020-01-03

**Authors:** Jesus Sanchez-Gomez, Jorge Gallego-Madrid, Ramon Sanchez-Iborra, Jose Santa, Antonio F. Skarmeta

**Affiliations:** 1 Department of Information and Communications Engineering, Faculty of Computer Science, University of Murcia, 30100 Murcia, Spain; jorgegm@um.es (J.G.-M.); skarmeta@um.es (A.F.S.); 2 Department of Electronics, Computer Technology, and Projects, Technical University of Cartagena, Cartagena, 30202 Murcia, Spain; jose.santa@upct.es

**Keywords:** LPWAN, SCHC, compression, fragmentation, LoRaWAN

## Abstract

The dawn of the Internet of Things (IoT) paradigm has brought about a series of novel services never imagined until recently. However, certain deployments such as those employing Low-Power Wide-Area Network (LPWAN)-based technologies may present severe network restrictions in terms of throughput and supported packet length. This situation prompts the isolation of LPWAN systems on islands with limited interoperability with the Internet. For that reason, the IETF’s *LPWAN* working group has proposed a Static Context Header Compression (SCHC) scheme that permits compression and fragmentation of and IPv6/UDP/CoAP packets with the aim of making them suitable for transmission over the restricted links of LPWANs. Given the impact that such a solution can have in many IoT scenarios, this paper addresses its real evaluation in terms not only of latency and delivery ratio improvements, as a consequence of different compression and fragmentation levels, but also of the overhead in end node resources and useful payload sent per fragment. This has been carried out with the implementation of middleware and using a real testbed implementation of a LoRaWAN-to-IPv6 architecture together with a publish/subscribe broker for CoAP. The attained results show the advantages of SCHC, and sustain discussion regarding the impact of different SCHC and LoRaWAN configurations on the performance. It is highlighted that necessary end node resources are low as compared to the benefit of delivering long IPv6 packets over the LPWAN links. In turn, fragmentation can impose a lack of efficiency in terms of data and energy and, hence, a cross-layer solution is needed in order to obtain the best throughput of the network.

## 1. Introduction

The Internet of Things (IoT) is a new paradigm that has permitted the creation of new business models by allowing the interconnection of end nodes with both power and computing constraints to the infrastructure. The applications of IoT include, but are not limited to, manufacturing, agriculture, energy management, environmental monitoring, building and home automation, healthcare, and transportation [1]. However, defining the hard edges of the IoT ecosystem is a complex task. One attempt to clearly define the IoT is presented in [2], where different definitions coming from relevant organization and standardization bodies are reviewed. It is concluded that the IoT is indeed a highly heterogeneous ecosystem with prominent connectivity needs for end devices. Accordingly, every end node composing an IoT system should be able to connect to the Internet to interact with the outside world. Hence, the device may be accessible from any other device no matter what communication technologies they employ. As a result, this paradigm has created a global decentralized network of machines equipped with data-gathering capabilities and different connectivity options [3].

Low-Power Wide-Area Network (LPWAN) is a new model that is gaining momentum to carry out the connectivity of constrained devices [4,5,6]. This technology notably extends the range of common Wireless Sensor Network (WSN)-based communication technologies available until now, such as ZigBee or IPv6 over Low-power Wireless Personal Area Network (LoWPAN). Solutions based on LPWAN have shown transmission distances over 15 km in rural areas and 6 km in urban environments [7]. Moreover, these systems offer high scalability, power efficiency, and reduced cost of end devices. They have proven to be a better fit for large WSN deployments than low-power short-range technologies or cellular alternatives [4,8].

Unlicensed LPWANs make use of a similar topology to cellular networks in the sense that devices directly communicate with base stations, i.e., these systems adopt a star or star-of-stars topology. The base station is a non-constrained element, designed to offer connectivity to a high number of end devices within the same cell, hence ensuring the scalability of these highly crowded networks. However, the LPWAN technology presents important limitations regarding its data-transmission capacity: (i) Layer 2 (L2) payload size from tens to hundreds of bytes; (ii) data rates from 10 to 100 kbps; and (iii) duty-cycle restrictions due to the use of unlicensed frequency channels, such as those in the Industrial, Scientific and Medical (ISM) radio band. For these reasons, LPWAN systems usually do not offer direct connectivity of end devices to the Internet. The main issue is related to the tiny L2 Maximum Transmission Unit (MTU) compared to the IPv6 MTU of 1280 bytes. The IPv6 protocol was designed to allocate addresses for all the nodes connected to the Internet with a header overhead of 40 bytes. This way, a single transmission of one IPv6 packet would need several fragments only for sending the IPv6 header, which is a big impediment for connecting IoT-based constrained nodes to the Internet. To solve the previous issues, an adaptation layer with header compression and packet fragmentation is needed. Following this path, the Internet Engineering Task Force (IETF) IPv6 over Low-Power Wide-Area Networks (LPWAN) workgroup has developed the Static Context Header Compression (SCHC), a new compression and fragmentation scheme for sending IPv6/UDP packets over LPWANs [9]. Thus, SCHC has been specifically designed for supporting IPv6 networking on LPWAN systems.

From a higher-layer perspective, the Constrained Application Protocol (CoAP) [10] has been chosen by the cited work group to be included on top of IPv6 and UDP, in order to enable application-layer communications in constrained scenarios. Its similarity with the REST architecture makes it easy to implement and to integrate within the Internet, since CoAP and HTTP use the same address space, caching mechanism and methods. Moreover, publish/subscribe (pub/sub) protocols have become relevant for data-oriented communications, where the client–server model is not appropriate because of its potential lack of reliability, scalability and flexibility, due to the massive number of end devices in IoT deployments. For that resaon, a pub/sub broker for CoAP is being defined by the Constrained RESTful Environments (core) IETF workgroup [11]. This scheme enables store-and-forward messaging between two or more nodes and facilitates data-retrieving from end devices with limited reachability. As with IPv6, the overload that the CoAP headers introduce in the restricted LPWAN links makes it unfeasible to be integrated directly. For this reason, another effort of the IETF’s LPWAN work group was to encapsulate CoAP over UDP/IPv6 [12] by employing the aforementioned SCHC mechanism.

It has been checked that these on-going standardization activities are not supported for the moment by proper implementations and evaluations in the literature, as it is later discussed. Moreover, we understand that analyzing the impact of compression and fragmentation of SCHC in real deployments can be of essential help for both researchers and engineers in the area. Hence, in this work, we deal with the issue of sending IPv6 packets over LPWAN technologies by implementing the SCHC mechanism and evaluating its operation on a real LoRa Wide-Area Network (LoRaWAN) deployment. In this set-up, end devices can directly communicate with any other host connected to the Internet by employing standardized network protocols, e.g., IPv6. Therefore, the main contributions of this work are the following:

- Exploration of the potential solutions for providing constrained end devices with an Internet connection, surveying available proposals.
- Development of the IETF SCHC scheme to integrate LPWAN networks within the Internet.
- Deployment of a real-life architecture using SCHC with LoRaWAN and considering both regular IPv6 and CoAP traffic.
- Analysis of different compression and fragmentation configurations to allow efficient transmission of packets in a particular LPWAN technology.
- Assessment of computational, memory and network overhead implied by SCHC.
- Evaluation of network benefits in terms of delay and packet delivery ratios.

The rest of the work is structured as follows: Section 2 contains a review of the different approaches to integrate IPv6 in constrained networks. On Section 3 the LoRaWAN technology is introduced. Section 4 presents SCHC, a state-of-the-art header compression and fragmentation scheme developed by the Internet Engineering Task Force (IETF). Section 5 contains a description of the proposed architecture and the real-life scenario employed to test its validity, as well as the implementation of the adaptation layer. Section 6 showcases the experiments’ results attained. Finally, Section 7 concludes the paper and draws future research lines.

## 2. Related Work

This section first approaches the problem of header compression and then presents contributions that support IPv6 connectivity in LPWANs. Finally, the focus is on the proposals in the field’s literature regarding packet fragmentation for constrained environments.

### 2.1. Header Compression Schemes

Header compression schemes are broadly divided in three families, namely: stateless, stateful, and hybrid. Stateless compression does not require each end to maintain settings about how to proceed with data packets. The header is compressed based on constant or commonly used values, as well as cross-layer information. Yet, this kind of compression schemes might not be very efficient in case of unpredictable traffic. On the other hand, stateful schemes build contexts if an inefficient compression is detected. These contexts are created locally and must be shared somehow with the receiving end to rebuild the original packet. Thus, this process consumes memory, processor time, and forces both ends of the compression to be synchronized. Consequently, stateful schemes present an overhead in power consumption by using radio communications only to maintain the context information. However, a high compression factor can be achieved, even with multiple flows simultaneously.

Aimed at sending IPv6 packets over WSNs, the IETF defined the 6LoWPAN technology in the RFC 4919 [13], which is specifically designed for IEEE 802.15.4-based networks. RFC 4944 [14] defines the packet format for 6LoWPAN and also a stateless compression mechanism that is limited. In the best-case scenario, it compresses the IP header down to 2 bytes, while the UDP header needs at least 4 bytes. In turn, the proposal in RFC 6282 [15] improves the compression factor, but it requires stateful context creation. Robust Header Compression (RoHC) [16] can provide a greater compression factor. However, the stateful nature of the scheme forces the use of radio messages to synchronize both ends of the link, and the its implementation complexity increases the processor, memory overhead and, thus, the overall power consumption of devices.

These adaptation layers have been tested to analyze their impact when transmitting messages larger than their MTUs. With regards to 6LoWPAN, authors in [17] compared different routing solutions in a real testbed with several devices, finding fragmentation useful to reduce retransmissions. Additionally, the work in [18] analyzed the impact CoAP Block-wise Transfers [19] in comparison with 6LoWPAN fragmentation. The results indicate that latency and reliability are affected differently. Also, the work in [20] showcased that compression and fragmentation may improve energy consumption of end devices up to 3%.

LPWANs use highly constrained radio links with very limited packet sizes. Such limitations imply that only a few bytes can be destined to the compressed headers of the network and transport layer. The aforementioned solutions have good performance for local network messages, but cannot provide headers shorter than 6 bytes after compression. As is later detailed, our solution bets on SCHC, which is a hybrid compression mechanism especially designed for LPWAN that takes the most of stateless and stateful approaches.

### 2.2. Adaptation Layer

Regarding the transmission of IPv6 packets over LPWANs, there are several works in the literature with proposals inspired in the 6LoWPAN compression mechanisms. In [21] the network architecture switches to different configurations depending on the type of processed packets. Hence, end devices can send either regular LoRaWAN or customized IPv6 packets to the gateway. This solution employs the gateway itself as an IPv6 router; consequently, every gateway must be modified in each particular case to work with the customized IPv6 packet. Another approach is presented in [22], where a LoRa radio chip is employed to support the 6LoWPAN stack. Thus, the Medium Access Control (MAC) layer behaves as a regular 6LoWPAN implementation, but it works over a different PHY technology, which may not be the most optimum solution due to the restrictions posed by LoRa.

It is important to note that the previous solutions found in the related literature do not follow the typical star-of-stars architecture proposed for LPWANs. Therefore, they force the modification of the gateways to employ them as network routers. Our work presents a deployment that is compliant with LoRaWAN specifications and embeds the message compression and fragmentation scheme currently under discussion within IETF’s lpwan work group.

The work in [23] evaluates SCHC against 6LoWPAN compression, assessing the improvement in compression rate over LoRaWAN, and it proposes a slight modification of SCHC to save memory in the storage of compression contexts. The same authors present in [24] another improvement for SCHC when a set of correspondent nodes in the Internet access an LPWAN device, or when the external access is performed from a node changing its network attachment point. This would imply, for instance, that the source IPv6 address and port could not be compressed. The work proposes the usage of dummy mappings to support a set of connection bindings under the same context rule with the aim of allowing a higher compression level. The key drawbacks of these two works are that first, regarding deployment and tests, network performance is theoretically extracted using time-of-air calculations and, secondly, from the base analysis of the solutions, the fragmentation capacities of SCHC are neither considered nor evaluated.

In our previous works [25,26], we implemented a preliminary IPv6/ICMPv6 adaptation layer based on SCHC that was validated in a real-life testbed. The end-device included a LoRa radio chip and was placed onboard a vehicle. The LoRa device continuously transmitted ICMPv6 Ping messages to a base station that decompressed the packets and relayed the Ping messages to their destination.

Work presented in [27] applied SCHC fragmentation over a LoRa network. Additionally, it tried to identify the impact of header compression and packet fragmentation, even if they initially fit the LoRaWAN’s MTU. The work analyzed the reduction of collisions among terminals transmitting at the same time, as the packet size is smaller, and concluded that after splitting the original packet to a certain number of fragments, the throughput converges. Hence, reducing the size of fragments even further does not improve the overall performance of the network, due to the increase of energy consumption of the system because of network overhead, presenting a clear disadvantage. As compared with our work, the contribution does not analyze the trade-off between fragmentation and LPWAN transmission settings, omits the LoRaWAN MAC layer by using only LoRa as PHY layer, and has the additional drawback of neither using a real implementation and deployment nor performing real tests.

In comparison with the cited works, we focus our contribution in the real evaluation of SCHC in terms of latency and delivery rate improvement, network overhead, and impact on end-device resources. We have evolved our initial and partial implementation presented in [25,26], which was a preliminary proof of concept, and deal with a whole SCHC middleware development evaluated on an off-the-self LoRaWAN-based deployment. It does not use alternative solutions such as those employing 6LoWPAN-based compression/fragmentation schemes, but we have followed the guidelines given by the IETF’s *lpwan* workgroup, respecting the original LPWAN’s principles. To the authors’ knowledge, this is the first SCHC implementation over real equipment evaluated in the literature considering both compression and fragmentation of the IPv6/UDP/CoAP stack, and analyzing the introduced overhead as well. With this solution, the reachability of the LPWAN end devices from the rest of the IoT nodes or services connected to the Internet is enabled. The deployed architecture and the developed algorithms are comprehensively detailed in the next sections.

## 3. LoRaWAN

LoRaWAN [28] is a long-range low-power wide-area network technology defined by the LoRaWAN Alliance in an open specification [29]. It is supported by first-line companies such as Cisco, Semtech and IBM, among others. The PHY layer of LoRaWAN uses LoRa [28,30], which is a proprietary radio technology owned by Semtech that employs Chirp Spread Spectrum (CSS) modulation. LoRa offers low power consumption to end devices while achieving a longer communication range than other low-power technologies such as Frequency Shift Keying (FSK) [8]. The key features of LoRa include:

- Long range. Connectivity of devices up to 20 km in rural areas and penetration of obstacles in urban settings, with ranges up to 6 km in these challenging scenarios.
- Low power. End devices’ battery lifetime may last up to 10 years [4].
- Mobility. Thanks to the CSS scheme, the decoding of the signal is possible even if the end-device is moving.
- Low cost. The price of radio modules is highly affordable.
- High capacity. The base-station radio chip can decode several transmissions in different radio channels at the same time, which increases network scalability.

Moreover, the user may configure different LoRa operational options according to their needs. The most relevant parameter is the Data Rate (DR). Lower DR values improve transmission robustness by increasing receiver sensitivity at the expense of bit rate. On the contrary, by increasingDR, throughput is greatly enhanced, but signal needs to be received at higher power level for its proper decoding. Other configurable parameters are bandwidth (BW), with a typical value of 125 kHz, and Coding Rate (CR), which permits the inclusion of redundant data within LoRa frames in order to enable error detection and correction.

The MAC layer proposal of LoRaWAN employs the unslotted ALOHA Protocol [31], hence the packets are transmitted at arbitrary times without performing any Clear Channel Assessment (CCA). This strategy lacks collision avoidance mechanisms for simplicity and cost reduction. To improve security, LoRaWAN includes a cryptographic suite-based on Advanced Encryption Standard (AES). This secures transmission at different OSI levels. The gateway is in charge of LoRA communication through the constrained link and, after receiving packets, it encapsulates LoRaWAN data messages and forward them to the Network Server (NS) via some backhaul network, e.g., 3G, 4G, Ethernet, or Wi-Fi. The NS is the central node, where all the intelligence and complexity resides.

In LoRaWAN, end devices are not associated with a specific gateway; hence, if the device is moving, there is no specific handover mechanism. Instead, the end-device follows a join procedure that connects the end-device to the network by communicating with the NS, which gives the end-device a unique network identifier. Observe that assigning a permanent IPv6 to the end node ensures the continuity and interoperability of the transactions while the join process is performed.

Finally, recall that LoRaWAN certifies three different device classes, namely Class A, B, and C. Class A (All) devices are sleeping all the time and only wake up to transmit a data message a few times a day. Two receiving windows are open immediately after each uplink transmission, then the device goes to sleep again. This class is focused on power efficiency and it is intended for battery-powered sensor devices. Class B (Beacon) is oriented to be used by actuator nodes. It implements all the functionality of Class A, and opens periodic receiving windows to allow the reception of downlink messages. Class C (Continuous) devices are continuously listening for receiving messages, hence this class is oriented to devices without power constraints.

## 4. Static Context Header Compression

As stated before, the IETF’s lpwan workgroup has developed SCHC [9], a compression and fragmentation scheme for sending IPv6/UDP packets over LPWANs, considering the compression of CoAP as well [12]. This proposal takes the advantages of both stateless and stateful compression. This way, both ends of the channel share a common pre-provided context that stores the header information. This context is static and does not change over the lifetime of the device, hence avoiding complex re-synchronization procedures. However, it also supports different compression configurations included in the pre-provided contexts, which are chosen according to a rule ID included in the compressed packets.

**Figure 1:**
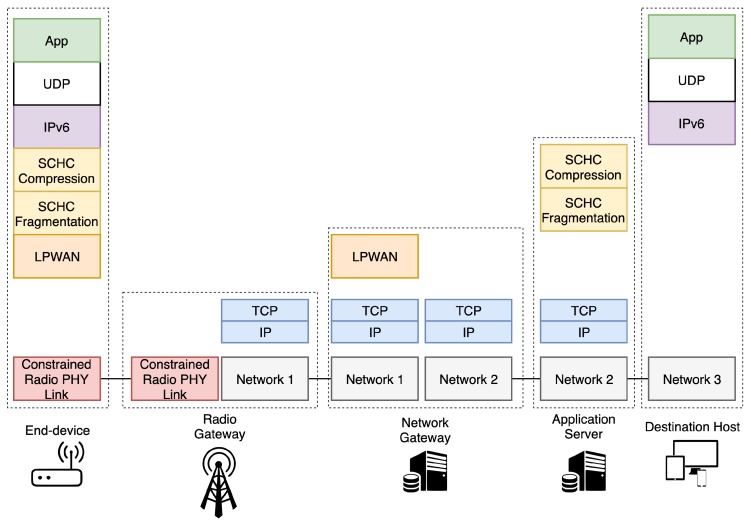
SCHC adaptation layer network stack over LPWAN.

SCHC works with the premise that devices embed built-in applications and that LPWAN traffic is highly predictable, i.e., the network manager knows in advance what kind of traffic is expected. Therefore, SCHC is an agnostic adaptation layer between IPv6/UDP/CoAP layers and the LPWAN technology. It is split in two different modules, namely the compression and fragmentation layers. An overview of the protocol stack is presented in Figure 1.

Regarding the SCHC compression scheme, the rule ID embedded in the packets avoids sending known header’s field values. This identifier represents a rule that provides the closest match to the original packet values. In other words, when the fields of a determined header are known by both ends, it is only necessary to send the rule ID together with the payload of the packet. If it is not possible to find a rule that matches any field in the packet, it must be sent without compression. Thus, the transmitted SCHC packet is composed of (i) the compressed header with a rule ID and a compression residue; and (ii) the payload from the upper layer packet. The rule ID identifies the rule used to compress the original packet while the compression residue is the rest of the header fields whose values are not defined in the applied rule. This residue may be absent in the case that the fields of the header are fixed and known in the specific applied compression rule.

In turn, the fragmentation procedure is applied when the SCHC packet does not fit within the LPWAN’s MTU. SCHC fragmentation works under the assumptions that data packets will not be delivered out-of-sequence and that the size of the fragment will not vary during the the transmission. Both assumptions significantly reduce the complexity of the reassembly process and the size of the fragmentation header. Each fragment is composed by a rule ID, a fragmentation header and the fragment payload. The fragment payload contains slices of the SCHC packet; consequently, a fragment payload may contain compression residue, application payload, or both.

Following this scheme (Figure 1), when an IPv6 packet must be transmitted, first the SCHC compression is applied, obtaining the SCHC packet. If the SCHC packet size is larger than the LPWAN’s MTU, then the fragmentation process is applied to the packet. Thereafter, the other end of the communication must apply the correspondent reassembly procedure and decompression context to obtain the original IPv6 packet. This process is bidirectional and may be initiated by both ends of the communication, i.e., the end-device or the host connected to an IPv6 network (e.g., the Internet). As mentioned in the previous sections, SCHC enables the direct interconnection of the previously isolated end devices integrated in LPWAN islands with the IPv6 ecosystem.

## 5. Architecture and Testbed

### 5.1. Architecture

**Figure 2:**
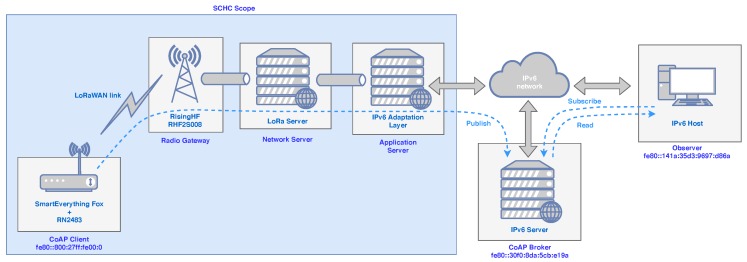
Deployed architecture for interconnecting the LPWAN segment with the IPv6 Internet.

A real architecture following the guidelines of the IETF’s lpwan workgroup has been defined and deployed as depicted in Figure 2. The objective of this deployment is validating and evaluating the performance of the SCHC scheme for providing IPv6 connectivity to a LoRaWAN network, whose motes use CoAP as application level protocol in a pub/sub fashion. As observed in Figure 2, the IPv6 adaptation module has been developed as a block independent from the rest of the elements composing the architecture. This modularity enables the agile management of SCHC contexts or the future integration of the implemented block within other LPWAN architectures.

The presented architecture consists of the next main blocks:

- LPWAN system, which is distributed among the transceiver used in the final device (a mote in our case), the gateway, the network server and the application server.
- SCHC Fragmentation/Reassembly middleware, which works at both the final device and the application server.
- SCHC Compression/Decompression (C/D) middleware, which also works end-to-end between the end-device and the application server.
- IPv6 adaptation layer, located exclusively at the application server. It is in charge of capturing IPv6/UDP packets or building them.
- Pub/sub service, which enables the information exchange between the end-device and the subscribed applications through a state-of-the-art CoAP broker [11] placed in the IPv6 cloud.

The protocol stacks used in the different nodes of Figure 2 are the ones depicted in Figure 1, being important to remark that the physical layer is provided by the hardware testbed we have deployed, while the SCHC middleware for fragmentation and compression has been implemented on the basis of the IETF draft [9].

Although we have used LoRaWAN, the architecture has been designed in a way that enables the operation of the system with any LPWAN solution, making the proposal independent from the underlying transmission technology. Following the SCHC draft, the SCHC C/D must be present in both the end-device and the connection point of the LPWAN system with the IPv6 network, which in our case is the application server (see Figure 1). With this configuration, the mote can directly send and receive SCHC packets in a transparent way for the application layer.

### 5.2. Implementation of the Testbed

To validate the proposed solution, a testbed has been deployed. The equipment details are also included in Figure 2. The real deployment consisted of one LoRaWAN Class A end-device, a base station, both LoRaWAN Network and Application servers, a CoAP broker to receive the IPv6 packets from the end-device and, finally, an application accessing to this broker through the Internet. This application was subscribed to the end-device’s updates and received the data from the broker, which had a certain topic already created. Following the proposed architecture, the end-device communicated with an IPv6 node (CoAP broker) through the LPWAN constrained radio link (LoRa) over the 868 MHz European ISM radio band. The CoAP broker was connected to the Internet, hence our compression and fragmentation solution acted as a middle layer between LPWAN and IPv6.

The end-device was composed by an Arduino-compatible microcontroller board (SmartEverything Fox), together with a RN2483 radio module by Microchip, which is a LoRaWAN Class A certified product for the 868 MHz ISM radio band. The radio module was connected to an omnidirectional 2.2 dBi antenna and it was configured to transmit at the maximum permitted output power (14 dBm). The base station employed was the RisingHF RHF2S008 model. It uses the Semtech’s SX1301 chip to communicate with the end devices. It was connected to an omnidirectional antenna of 5 dBi gain. All the LoRaWAN packets received by the base station were forwarded to the network server (Figure 2). Finally, regarding the configuration of the LoRa parameters in the end-device, the employed bandwidth was 125 kHz, the data rate was set to the values of DR5, DR3 and DR0, and the CR was constantly set to a value of 4/5.

The SCHC middleware is based on the initial development presented in [25,26]. It has been implemented in C and supports IPv6, UDP and CoAP compression. As a difference with recent implementations, such as the one in [32], it fully supports compression and fragmentation.

### 5.3. Set-Up of SCHC Compression and Fragmentation

Three different compression levels have been considered in our implementation in order to make CoAP packets suitable to be transmitted over the constrained radio link, namely (i) non-compression (original packet); (ii) IPv6/UDP compression, where both the IPv6 and UDP headers are removed from the original IPv6 packet and are substituted by a Rule ID (1 Byte), followed by the uncompressed CoAP header (15 bytes); and (iii) IPv6/UDP/CoAP compression, in which the IPv6, UDP and CoAP headers are substituted by the corresponding Rule ID (1 Byte) and a compression residue (4 bytes).

In the case of IPv6/UDP compression, the SCHC rule applies the compression action for all the header fields. This is possible thanks to knowing in advance the IPv6 addresses and UDP ports of both ends. This way, the 40 bytes of the IPv6 Header, and the 8 bytes of the UDP header are compressed down to 0 bytes, hence just the Rule ID of 1 byte is sent.

In turn, to achieve the full stack compression (IPv6/UDP/CoAP), the CoAP header is reduced to 4 bytes. These bytes correspond to the *Message ID* and *Token* fields that may vary between messages. The IPv6 and UDP headers can be predicted, as indicated in the previous case.

To apply fragmentation to the original CoAP messages, the next steps are followed. First, the end-device compresses the original IPv6 packet following the previous IPv6/UDP/CoAP procedure. Next, the SCHC packet is split into different-size SCHC fragments. Finally, the SCHC fragments are sequentially sent as regular LoRaWAN messages through the radio link. Each fragment contains the Rule ID (1 Byte), a Fragment Compression Number (FCN) (1 Byte), and the fragment payload. The fragment payload contains data from the original SCHC packet.

Please note that in our implementation, the SCHC Rule ID is sent in the LoRaWAN’s fPort field (see [29] for details), as this field may be configured by the user. Thus, the byte corresponding to the Rule ID of all fragments and packets is embedded within the LoRaWAN’s 13 bytes-length header instead of being transmitted within the SCHC header field set by default. With this strategy, we save one additional byte, avoiding increasing the total length of the LoRaWAN message due to this header field. Moreover, as the SCHC packets are transmitted sequentially, the identifier of the original fragmented packet (Datagram Tag—*DTag*—in the specification [9]) is not sent for further reducing the overhead.

The SCHC fragmentation reliability mode employed in this proposal is No-ACK [9], hence, no SCHC special acknowledge packets are generated by the Fragmentation/Reassembly layer. Therefore, the SCHC mechanism relies on the LoRaWAN network for the retransmission and confirmation of data messages. Under this configuration, the end-device transmits all the LoRaWAN messages marked as *confirmed*, which forces the LoRaWAN’s network server to independently acknowledge each uplink packet by transmitting a downlink ACK packet. The end-device keeps retransmitting each fragment until the confirmation from the LoRaWAN’s network server is received. In that case, the end-device starts the transmission of the following fragment, until all fragments are successfully delivered.

### 5.4. Testing Scenario

**Figure 3:**
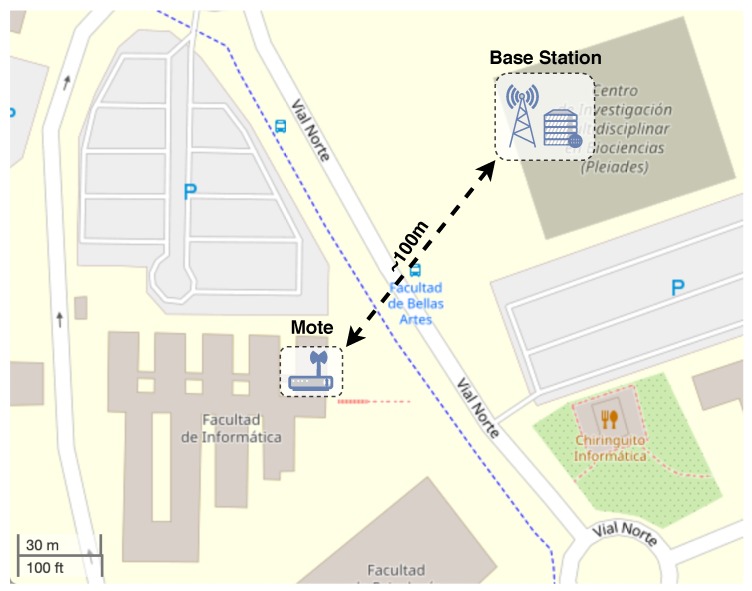
Testing scenario at the University of Murcia campus (38.023812 N, −1.173500 E).

Two real scenarios considering both best-case and challenging conditions have been evaluated to explore the system with different levels of adversity for radio communications. The best-case scenario comprised an indoor test inside a laboratory at the University of Murcia. The mote and the base station were placed next to each other. This scenario was employed to have a reference under ideal conditions. On the other hand, in the challenging scenario the mote was placed in a different building, 100 m away from the base station with no line of sight, blocked by many walls between both elements. This scenario is showed in Figure 3, indicating the position of the mote and the LoRaWAN gateway. It is a challenging deployment, as it provoked a notable level of packet losses. The aim of considering this scenario is to study the benefits of employing different LoRa or SCHC configurations with no ideal links.

During the tests, the end-device periodically published to the topic */storage* using CoAP PUT messages with the payload containing an integer with the temperature measured by an embedded sensor. The transmission rate of such messages was 1 message every 140 s to be compliant with the duty cycle of the 868 MHz ISM band. The CoAP broker answered with a *2.04 Changed* message, which also acted as an acknowledgement in this transaction. We assume that the CoAP Simple Discovery process has been performed before, thus the broker’s presence and availability is known by the end-device. Globally, the entire experiment was divided into five independent sessions for each scenario, in which 100 consecutive transmissions from the end-device to the broker were made for each configuration. Thus, the results shown in the next Section represent the average value extracted from the five sessions. Although only one end-device is used in the tests, the testbed is fully representative of the kind of evaluation needed to validate and analyze the performance of the compression and fragmentation mechanisms. Previous studies such us our work in [4], discuss the high scalability features of LPWANs, which is further guaranteed by duty-cycle restrictions imposed by regulatory bodies. In our case, we have considered the European regulations, i.e., EU863–870MHz ISM Band [33], which mandate a maximum channel occupancy (duty cycle) of 1% of the time for the 868 MHz ISM band.

For our compression tests, as stated above, three levels of compression were applied to CoAP packets of 111 bytes (original IPv6 packet length). As a result of applying the different compression levels, the obtained packet sizes were:

- No compression: 111 bytes (100%).
- IPv6/UDP compression: 62 bytes (55%).
- IPv6/UDP/CoAP compression: 51 bytes (46%).

Table 1 includes the details of the packets sent using different DR configurations. It is important to see that the original packet (111 bytes) and the one with IPv6/UDP compression (62 bytes) exceed the maximum Time-on-Air (ToA) imposed by the frequency band’s duty cycle for DR0 (rows in bold). Therefore, at first, these packets would not be authorized to be sent by using this DR if no compression is considered. However, by applying the IPv6/UDP/CoAP compression with the SCHC scheme, the packet length is now valid for this DR (DR0), while the payload content remains unmodified.

For the fragmentation tests, large IPv6 packets of 1156 bytes were considered, which, after the application of IPV6/UDP/CoAP compression, resulted in 1093 bytes. After adding 1 byte for the Rule ID and a Compression Residue of 4 bytes, the final SCHC packet was 1098 bytes length. Then, three different fragment sizes were employed to test the performance of these levels of fragmentation: high, medium, and low.

The associated fragment sizes to each fragmentation level are presented in Table 2. The column *PHY Length* shows the total length of the LoRa packet, including 13 bytes of LoRaWAN protocol header and 1 Byte of overhead, because of the fragmentation strategy employed. The column *Fragment Pay.* shows the amount of bytes that belong to the 1093 Byte IPv6 packet contained in the LoRaWAN packet. The column *ToA* refers to the ToA of the packet. Please note, that as aforementioned, in this particular implementation of SCHC, the Rule ID (1 byte) is embedded in the LoRaWAN header as the fPort field. Thus, it is not included to compute the total PHY packet length.

### 5.5. Performance Evaluation Metrics

As shown above, the base ToA calculation for each DR is used as a reference of the delay in the wireless communication link. Since LoRaWAN is a non-contention-based network, theoretical ToAs are good estimation of the real delay of packets correctly delivered. This base value has been also used, together with the time needed by the end-device in the compression and fragmentation, to compute the overall delay resulted in the solution. Moreover, the Packet Delivery Ratio (PDR) has been found of great importance to evaluate the robustness of the radio link. Considering fragmentation and compression issues, the lower the payload of the data message, the higher the overhead due to the LoRaWAN headers and the SCHC fragmentation. Thus, the PDR takes an important place in measuring the performance of the chosen configuration. For example, if the size of the data message is high but the PDR is poor, the overall performance is reduced, so a lower packet size may yield better results.

In addition, to measure the performance of the different configurations and packet sizes employed, a performance metric called GoodputperToA has been defined aiming at measuring both the data-delivery and energy efficiency of the different configurations while performing the data transmissions. This metric represents the amount of payload bytes (non-overhead data) that is transmitted during the ToA needed by a message transmission. As the ToA of each transmission is directly related with the energy consumption of the end-device, this metric indicates the efficiency provided by the chosen configuration for transmitting certain amount data (1).

(1) $$Goodput\;per\;ToA=\frac{P\_Confirmed}{P\_Transmitted}\;\cdot \;\frac{Useful\;Payload}{Time\;on\;Air}$$

P_Confirmed is the number of packets received and confirmed while P_Transmitted is the number of packets transmitted. Please note that this part of the equation represents the PDR. UsefulPayload is the useful amount of data transmitted by each packet, i.e., excluding headers (bits), and TimeonAir is the ToA of one packet measured in seconds. Please note that the second part of the equation is notably determined by the compression and fragmentation configurations. Thus, we obtain a bits-per-second metric that measures the system goodput in terms of ToA. Hence, the most profitable configuration will carry more useful bytes to the receiving end, while consuming the lowest amount of ToA and, consequently, battery energy.

Finally, with the aim of complementing the computational time needed by compression and fragmentation, we have also measured the memory usage of the SCHC middleware. The size of the different data structures necessary per compressing context has been measured to assess the feasibility of the solution for high-constrained devices.

## 6. Results and Discussion

The deployment described in the previous section was used to conduct three different studies. First, the performance obtained when varying the LoRa’s DR and the SCHC’s compression level without fragmentation is evaluated. Secondly, a discussion of the impact of applying different fragmentation strategies on the transmission of large CoAP packets is provided. Finally, the computational impact of the solution in the end-device is analyzed.

### 6.1. SCHC Compression Performance

As explained in Section 5, first a series of transmissions from the mote to the broker in a best-case scenario were performed. As expected, the results do not vary much in this case and are almost equal with each configuration, as can be seen in Table 3. It is important to note that all the uplink (UL) PDRs are 100%, which is the key traffic direction in sensing-oriented deployments. This behavior is explained by the proximity of the mote and the base station. The same happens with the downlink (DL) transmissions, although there were some packet losses, which are attributed to eventual and negligible local interference. Given that the experiments were conducted in a research and industrial area with many systems using the 868 MHz ISM band, we detected simultaneous transmissions in the same band that affected especially the reception rate. This effect was more evident in the DL channel because the reception sensibility of the end node presents a worse performance, given the antenna characteristics, as compared with the one used in the gateway.

Overall, the attained results were highly satisfactory, no matter the DR or the compression level considered. Observe in Table 3 some blank spaces that are related to the excessive length of the original and the IPv6/UDP-compressed packets, which exceeded the maximum message length permitted in LoRa’s DR0, as explained above.

The results collected from the more realistic-conditions scenario are clearly different from the ones previously presented, as shown in Table 4. However, this testbed permits us to evaluate the performance of the system in an adverse scenario when using different compression schemes and DRs. Therefore, observe in Table 4 that the general tendency is that PDR improves as the DR decreases and the compression is higher, obtaining the best results with DR0 and IPv6/UDP/CoAP compression.

In general, the results were better in the case of the uplink transmissions in comparison with the downlink ones. As said, the DL channel is affected by the lower gain of the antenna used in the end-device. Therefore, the base station was able to properly decode more received packets in comparison with the mote, which presents a more restricted hardware characteristics, e.g., sensitivity, for properly decoding the transmissions arriving at a low power level. This higher variability of the DL channel is evident in Table 4, when comparing the results obtained for the same DR, but using different compression modes. Nevertheless, the improvement in PDR is clear per row as DR changes, given that tests were consecutive performed for each compression mode and radio conditions were common.

**Figure 4:**
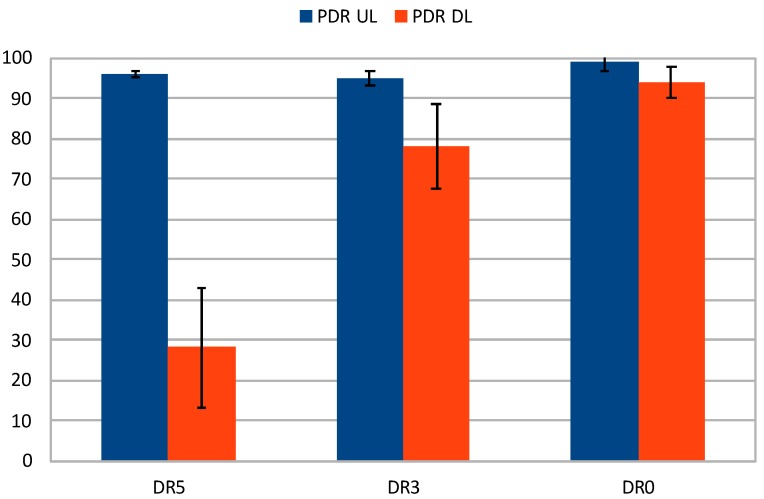
Uplink and downlink PDR with full compression.

Special attention must be paid to the results obtained with full compression (last row of Table 4), which are also plotted in Figure 4 including the confidence intervals (α=0.05). It can be seen how the downlink PDR increases significantly as the DR decreases, starting at 28% for DR5 and growing up to almost 94% for DR0. The uplink also increases, although more discretely, and the overall performance is highly satisfactory, with PDR figures over 95%. The better performance can be also noted by looking at the confidence intervals in the plot, which improve due to the greater robustness and link stability provided by lower DRs. Therefore, it is evident that a proper compression and DR configuration can overcome downlink transmission issues.

**Figure 5:**
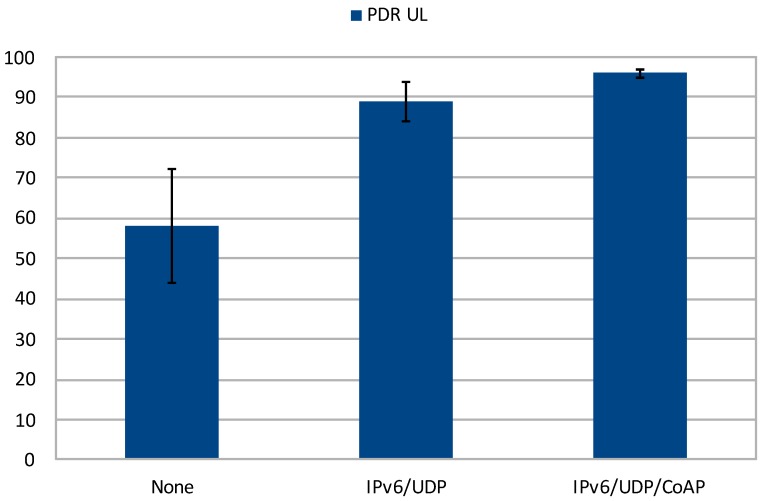
PDR evolution with DR5 and different levels of compression.

Regarding the evolution of the PDR by fixing the DR and applying different compression levels, the DR5 uplink results are very representative for the more realistic scenario (Figure 5). It can be seen how the reliability of the channel improves as the compression increases. This is due to the ToA reduction, as the packets spent less time in the air and the probability of errors is lower. This effect can be also inferred by observing the confidence intervals attained (α=0.05), which clearly decrease when applying greater levels of compression. These outcomes demonstrate that reducing the packet size is highly beneficial for the performance of the system while keeping the data rate untouched.

### 6.2. Fragmentation

The results attained from the tests involving fragmentation are presented in Table 5. The column *Dev Tx* represents the amount of LoRaWAN data messages transmitted by the radio module. The column *GW Rx* shows the amount of LoRaWAN messages successfully received by the radio gateway. The column *GW ACK Tx* shows the amount LoRaWAN ACK packets transmitted by the gateway. Please note that the gateway transmits one ACK packet for each successfully received packet from the device. Thus, the columns *GW Rx* and *GW ACK Tx* always have the same values. This information has been included in the table for the sake of clarity. The column *Dev ACK Rx* includes the number of LoRaWAN ACK packets received by the mote. The columns *PDR UL* and *PDR DL* represent the PDR for the uplink and downlink transmissions, respectively. The column *IPv6 packets sent* shows the number of IPv6 packets with a payload of 1093 bytes that were successfully transmitted by the device.

The results in Table 5 showcases a rise in the PDR for the uplink transmissions when the packet is small (greater level of fragmentation) and the DR is low. This is because the link becomes more robust and the reception of packets is improved. Concretely, the results for DR5 show a great difference in the uplink PDR when the packet size is reduced, passing from 18.86% up to 81.79%. This behavior is explained by the fact that DR5 is the least reliable data rate under study, so with this configuration the transmission is very susceptible to be affected by path loss or other environmental effects. Therefore, using shorter fragments leads to more reliable transmissions. On the other hand, DR3 and DR0 show less differences between the lowest and highest uplink PDR figures. This is due to DR3 and DR0 being more reliable LoRa configurations in comparison with DR5; even so, using small fragment sizes also demonstrates an improvement in the PDR results.

**Figure 6:**
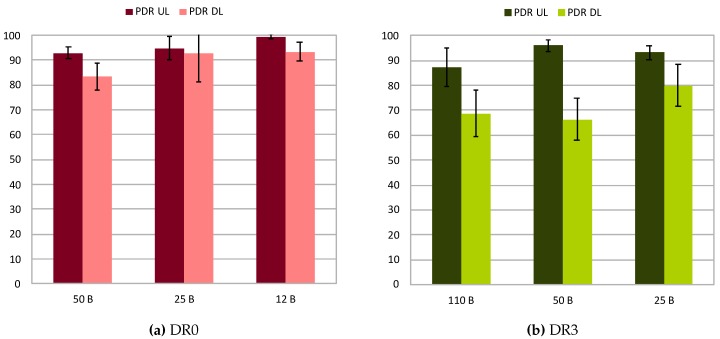
PDR attained in fragmentation tests for DR0 (**a**), and DR3 (**b**).

**Figure 7:**
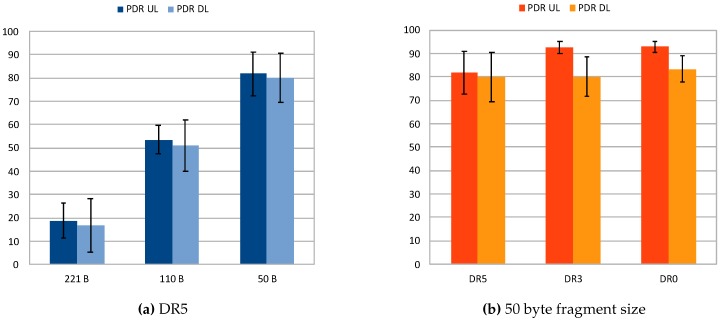
PDR attained in fragmentation tests for DR5 (**a**), and 50 byte fragment size (**b**).

For the sake of clarity, Figure 6a,b and Figure 7a showcases the PDR attained for both uplink and downlink for the different fragment sizes employed in each of the studied DR configurations. Observing them, the PDRs for the different DR configurations show a constant increase thanks to the effect of fragmentation when decreasing the size of packets. Considering these results, it can be stated that employing fragmentation helps to increase the reliability of transmissions. In turn, by fixing the size of the fragments to 50 bytes, the effect of LoRa configuration can be studied as shown in Figure 7b. It shows a moderate increase in the PDR when the DR is reduced, given extra robustness to the transmission with lower DRs. These results agree with those observed in the previous experiment, which confirm the superior performance of LoRa in terms of reliability when low DRs are employed.

**Figure 8:**
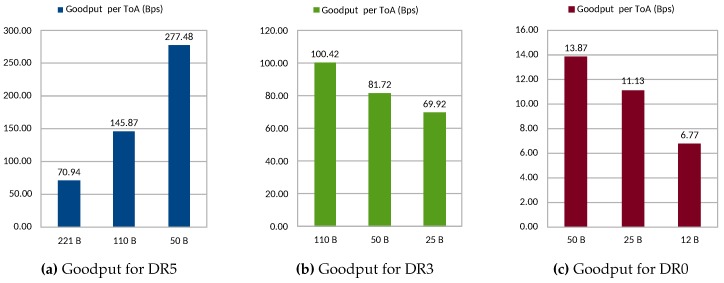
Goodput per ToA for the different fragment lengths with DR5 (**a**), DR3 (**b**), and DR0 (**c**).

Although the results discussed above would suggest the use of low DR and small fragments to reach the most robust configuration, there is a trade-off to be considered between this aspect and the network/energy efficiency. This fact is demonstrated by analyzing the results obtained by applying (1) to the data presented in Table 5. The resulting values are presented in Figure 8. Recall that this metric represents the efficiency of the transmission in terms of the number of useful bytes sent per second of ToA. This metric is also related to the energy efficiency of the end node, given that a longer duration of the transmissions (ToA) imply more power consumption by the radio module, besides a higher delay. Observe that DR5 configuration presents a high performance increase when reducing the packet size. This is due to the poor PDR results in terms of PDR for longer packet lengths such as 221 bytes and 110 bytes. For DR3 and DR0, the performance decreases with smaller fragments. This is explained by the diminished differences in PDR values, as shown in Table 5, and especially by the overhead introduced by employing small packets, which presents a disadvantage for these configurations. Observing the results presented in Table 5, concretely the last column, the effects of this behavior can be understood. By evaluating each DR configuration individually, observe how the number of complete IPv6 packets received after the fragmentation process increases as the fragment length is reduced for the case of DR5. On the contrary, for slower transmission configurations, namely DR3 and DR0, the best results are attained with the longest fragment length. Overall, the better configuration for the studied scenario is DR5 with a fragment length of 50 bytes, which yields the best global results with a reached goodput of 2.2 kbps. In general, given the results obtained, it is important to remark the aforementioned balance between obtaining a better PDR and the cost of higher fragmentation overhead.

### 6.3. End Node Overhead

As said above, the end-device used is a SmartEverything Fox board. The main features of this hardware are CPU Atmel D21 with 48 MHz of CPU clock speed, 256 KB of flash memory and 32 KB of SRAM. The board has been evaluated in a set of tests to check its performance when using the compressing and fragmentation mechanisms.

IPv6/UDP/CoAP compression and fragmentation have been configured in the firmware to cover a set of 216 tests, obtaining the results shown in Table 6. These include time figures and memory needed to maintain SCHC contexts in the device (confidence intervals with α=0.05). As the results reveal, the compression lasts one order of magnitude more than fragmentation in the operation of the middleware in the device. This is due to the fewer instructions needed in the process and, consequently, a lower number of CPU cycles. An overall figure of 7.91 ms is obtained when using both compression and fragmentation. This value fully justifies the application of our SCHC-compliant solution to reduce the time needed by the packets to reach the wired network. As detailed in Table 1, the ToA is reduced by 35% when applying IPv6/UDP compression, and an extra 10% more when using IPv6/UDP/CoAP compression. Hence, this great improvement can be obtained at the expense of an extra 7.91 ms of overhead, which may be negligible as compared with ToA values ranging from 205.06 ms (DR5) to 4759.55 ms (DR0) ms when no compression is used.

Regarding memory needs, it has been checked that each compression context maintained by our SCHC middleware needs 609 bytes in the worst case. Our particular Arduino device is provided with 32 KB of SRAM; hence, about 50 contexts could be maintained simultaneously.

From the results it can be extracted that even a quite constrained device such as the one used here is able to use our solution efficiently. As compared with other well-known platforms in the same segment, our hardware is equivalent to the one used by Arduino Nano, with the SAM D21 CPU at 48 MHz and 32 KB of SRAM. Although it is clearly below the features of the Raspberry Pi Zero, with an ARM1176 CPU at 1 GHz and 512 MB of SRAM, or the BeagleBoard PocketBeagle, with an Arm Cortex A8 CPU at 1 GHz and 512 MB of SRAM. This implies that even better performance could be easily obtained with other commercially available products with fewer power constraints. Hence, our evaluation can be understood as a baseline performance study.

### 6.4. Discussion

The presented results showed the benefits that the use of the SCHC mechanism provides to LPWAN networks. It has been proved that by using this scheme, IPv6/UDP/CoAP packets that were not able to be transmitted, now can be sent by applying the header reduction and decreasing the DR of the communication. Accordingly, an inherent issue should be mentioned, namely the extra resources needed by the algorithm, especially in the end node, and the notable increase of the ToA as the DR is reduced. Nevertheless, considering the results obtained, the advantages presented above make these two problems to be more than acceptable.

In addition, the use of CoAP and IPv6 in the motes enables their reachability from the Internet. The fragmentation further extends the compression possibilities of using larger packets, such as the ones involved with CoAP and IPv6. At the same time, the reliability of the channel can be improved by selecting the most proper fragment length. This is a great advantage, because with these mechanisms a host connected to the Internet can directly communicate with a mote located inside an LPWAN infrastructure. Under regular conditions in LPWAN deployments, if an external node needs to communicate with a mote, it has to establish a connection to the network server, which then forwards the message and receives the response through the specific LPWAN technology. Using SCHC, the application server applies the (de)compression, depending on the direction of the communication, and forwards the packet, making the LPWAN communication transparent to the external node. Furthermore, although it may seem obvious, a remarkable aspect of this system is the fact that the mote itself can initiate a connection to a server located in the Internet, which opens a variety of services linked to the IoT paradigm.

## 7. Conclusions

Constrained devices connected to LPWANs sustain rigorous restrictions when transmitting data over the radio link. These limitations forbid devices from directly connecting to the Internet through a standardized protocol such as IPv6. The overhead introduced by the IPv6 header (40 bytes), and the relatively large IPv6 MTU (1280 bytes) required, imposes the application of header compression and fragmentation of packets.

In this work, we have analyzed the challenge of bringing constrained devices, connected through LPWAN technologies, to the IoT global ecosystem. As there are not remarkable works related to Internet interconnection with LPWAN networks that consider real deployments and empirical evaluation, one of the main goals of this work has been to evaluate the behavior of the SCHC approximation attending the two main subsystems, i.e., compressing and fragmentation. This has been done with a full implementation of the middleware and the deployment of a LoRaWAN network completely functional within our university campus. The tests have been focused on analyzing the impact of (i) header compression; (ii) data rate configuration and (iii) fragmentation size, attending to the reliability of the channel, the overhead of the end node and the overall performance of the system in terms of transmission efficiency.

The results showed that the developed solution permits interconnection of constrained devices with a CoAP broker in the Internet through LPWAN technologies following a pub/sub scheme, and final hosts can consume data gathered through a subscription. The discussion highlighted an important improvement in the reliability of the LPWAN links when the data rate is smaller and, what is more relevant in this study, when compression is higher and packets are fragmented; however, there is a limitation regarding the overall gain due to the overhead imposed by the fragmentation headers. The processing time needed by end devices is below 8 ms, and the memory usage per SCHC is of only 609 bytes, with the great benefit of reducing the ToA in more than 40% when full compression is applied. Hence, it is necessary to consider especially a trade-off between fragment sizes, channel reliability, data bandwidth, and energy consumption, which will be dependent on the concrete application or deployment scenario.

Future study will consider the in-depth analysis of mobility when LPWAN technologies are used together with these mechanisms, and the application of an overall networking solution in smart city scenarios, considering transport monitoring and moving sensors. Our system is currently being improved with a generic key exchange mechanism for LPWANs, following the line initiated in [34].

## Figures and Tables

**Table 1:** ToA of the packets under consideration.

Compression Level	Packet Length (B)	DR	ToA (ms)	Max. ToA (ms)
No compression	111	DR5	205.06	399.61
		DR3	656.38	676.86
		**DR0**	**4759.55**	**2793.47**
IPv6/UDP	62	DR5	133.38	399.61
		DR3	431.10	676.86
		**DR0**	**3121.15**	**2793.47**
IPv6/UDP/CoAP	51	DR5	118.02	399.61
		DR3	390.14	676.86
		DR0	2793.47	2793.47

**Table 2:** Configuration parameters for the fragmentation tests.

Test Name	Data Rate	PHY Length (B)	Fragment Pay. (B)	ToA (ms)
DR5-221B	DR5	235	221	368.90
DR5-110B	DR5	124	110	205.06
DR5-50B	DR5	64	50	118.02
DR3-110B	DR3	124	110	656.38
DR3-50B	DR3	64	50	390.14
DR3-25B	DR3	39	25	267.26
DR0-50B	DR0	64	50	2793.47
DR0-25B	DR0	39	25	1974.27
DR0-12B	DR0	26	12	1646.59

**Table 3:** PDR attained in a best-case real testbed.

Data Rate	DR5 PDR (%)		DR3 PDR (%)		DR0 PDR (%)	
Compression/Dir	UL	DL	UL	DL	UL	DL
None	100	96	100	97		
IPv6/UDP	100	100	100	98		
IPv6/UDP/CoAP	100	96	100	96	100	98

**Table 4:** PDR attained in a challenging real testbed.

Data Rate	DR5 PDR (%)		DR3 PDR (%)		DR0 PDR (%)	
Compression/Dir	UL	DL	UL	DL	UL	DL
None	58	0	61	29.51		
IPv6/UDP	89	42.70	84	95.24		
IPv6/UDP/CoAP	96	28.13	95	77.89	99	93.94

**Table 5:** Fragmentation Test Results.

Test ID	Dev Tx	GW Rx	GW ACK Tx	Dev ACK Rx	PDR UL (%)	PDR DL (%)	IPv6 pkts. Sent
DR5-221B	228	43	43	27	18.86	16.79	5
DR5-110B	228	122	122	62	53.51	50.82	6
DR5-50B	313	256	256	205	81.79	80.08	9
DR3-110B	252	220	220	151	87.30	68.64	15
DR3-50B	276	265	265	176	92.66	80.01	8
DR3-25B	301	281	281	225	93.36	80.07	5
DR0-50B	169	157	157	131	92.90	83.44	5
DR0-25B	190	180	180	167	94.74	92.78	3
DR0-12B	198	197	197	184	99.49	93.40	2

**Table 6:** End node overhead.

Task	CPU cycles / Mean Time (ms)
Compression	343,518.2 ± 7.68/7.16 ± 0.16
Fragmentation	35,863.3 ± 9.12/0.75 ± 0.19
**Memory structure**	**Memory space (B)**
Compression context	609

## Abbreviations

The following abbreviations are used in this manuscript:

6LoWPAN	IPv6 over Low-Power Wireless Personal Area Network
AES	Advanced Encryption Standard
CCA	Clear Channel Assessment
CSS	Chirp Spread Spectrum
CoAP	Constrained Application Protocol
DR	Data Rate
FCN	Fragment Compression Number
IETF	Internet Engineering Task Force
ISM	Industrial, Scientific and Medical
IoT	Internet of Things
LPWAN	Low-Power Wide-Area Network
LoRaWAN	LoRa Wide-Area Network
MAC	Medium Access Control
MTU	Maximum Transmission Unit
NS	Network Server
PDR	Packet Delivery Ratio
pub/sub	publish/subscribe
RoHC	RObust Header Compression
SCHC	Static Context Header Compression
SME	SmartEverything Fox board
ToA	Time of Air
WSN	Wireless Sensor Network
